# Can the world’s favorite fruit, tomato, provide an effective biosynthetic chassis for high-value metabolites?

**DOI:** 10.1007/s00299-018-2283-8

**Published:** 2018-03-28

**Authors:** Yan Li, Hsihua Wang, Yang Zhang, Cathie Martin

**Affiliations:** 1 Key Laboratory of Bio-resource and Eco-environment of Ministry of Education, College of Life Sciences, Sichuan University, Chengdu, 610065 Sichuan People’s Republic of China; 2 Metabolic Biology Department, The John Innes Centre, Norwich Research Park, Norwich, NR4 7UH UK

**Keywords:** Tomato, Metabolic engineering, Specialized metabolites, Chassis, Scale-up production

## Abstract

Tomato has a relatively short growth cycle (fruit ready to pick within 65–85 days from planting) and a relatively high yield (the average for globe tomatoes is 3–9 kg fruit per plant rising to as much as 40 kg fruit per plant). Tomatoes also produce large amounts of important primary and secondary metabolites which can serve as intermediates or substrates for producing valuable new compounds. As a model crop, tomato already has a broad range of tools and resources available for biotechnological applications, either increased nutrients for health-promoting biofortified foods or as a production system for high-value compounds. These advantages make tomato an excellent chassis for the production of important metabolites. We summarize recent achievements in metabolic engineering of tomato and suggest new candidate metabolites which could be targets for metabolic engineering. We offer a scheme for how to establish tomato as a chassis for industrial-scale production of high-value metabolites.

## An important crop

Economically, tomato (*Solanum lycopersicum*) is the most important horticultural crop, and its production, by yield, is second only to potato, across the world (Peixoto et al. 2017). The short life cycle (90–120 days) and self-compatibility of tomato facilitate its cultivation as a cash crop for both small as well as large-scale growers. According to their different commercial uses, tomato varieties can be divided into fresh market varieties which are usually produced in greenhouses and processing varieties which are often field-grown, for industrial uses (Zsögön et al. 2017). Besides water and fertilizers, successful tomato production requires optimised cultivation methods and management, pest control, and appropriate post-harvest storage (Liu et al. 2017a, b). Under optimized conditions, tomato productivity can easily reach 20–50 tons per hectare (Wang and Seymour et al. 2017; Tieman et al. 2017). China and the United States are the two largest tomato-producing countries in the world (http://faostat3.fao.org/browse/Q/QC/E). In the US, in 2016, the total area under tomato cultivation was 364,800 acres and the total yield reached 16 million tons with a value of just over $2 billion (USDA 2017; Zsögön et al. 2017).

## An excellent crop model with substantial infrastructure as well as tools and resources for metabolic engineering

Tomato is consumed fresh or as a processed product in canned tomatoes, paste, puree, ketchup, juice and pasta sauces. Tomato consumption globally averages 20 kg per capita per annum, with the USA, China and Italy consuming double these levels, on average (http://www.agribenchmark.org). Tomato is the only fruit/culinary vegetable to have increased in consumption in the USA over the past 50 years.

Some metabolic engineering of tomatoes have been focused on its nutritional improvement as a food, and production of tomatoes enriched in nutrients may be the most cost-effective route to consumers for effective nutritional improvement without requiring substantial shifts in diet (Butelli et al. 2008; Scarano et al. 2017).

Alternatively, tomato can be used as an effective production platform for high-value compounds, such as drugs, and for such uses the primary objective is to extract and purify the high-value bioactives produced (Zhang et al. 2015).

Tomato has been an important model plant for biological research. The genome sequence of tomato has been published (Consortium 2012) and its epigenome and extensive resequencing data are available from the Sol Genomics Network (SGN: https://solgenomics.net) (Lin et al. 2014). The Tomato Genomics Resources Database (TGRD: http://59.163.192.91/tomato2/) houses RNA-seq and microarray data for tomato as well as some metabolite data. TGRD allows interactive browsing of tomato genes, micro RNAs, simple sequence repeats (SSRs), quantitative trait loci (QTL) and the Tomato-EXPEN 2000 genetic map (Suresh et al. 2014). There are extensive genetic resources in the form of well characterised mutant collections (Tomato Genetic Resource Center, TGRC University of California, Davis: http://tgrc.ucdavis.edu/), several excellent TILLING populations for mutant discovery (UC Davis in Heinz-1706; INRA Bordeaux in MicroTom, INRA Versailles in M82), Red Setter and Money Maker (phenotypes available through SGN and LycoTILL) and a phenotypic library of additional mutations catalogued in ‘The Genes that Make Tomato’ available through SGN. Recent progress in tomato metabolomics now provides substantial information about its primary and specialized metabolism and the pathways involved in synthesis and turnover (Luo 2015; Tieman et al. 2017; Zhu et al. 2018). Together with efficient genome editing tools (Brooks et al. 2014; Sprink et al. 2015; Soyk et al. 2017), these advantages make tomato an excellent choice for metabolic engineering. The community of scientists working on tomato is also exceptionally collaborative, with researchers exchanging mutants, accessions, genomics data and new protocols freely and constructively, prior to publication.

The conflict between demands for specific metabolite production and growth dependent on photosynthesis places limits on the levels of production of specialized metabolites possible in photosynthetic tissues, but fruit-specific production in tomato allows high productivity without yield penalties (Butelli et al. 2008; Luo et al. 2008; Zhang et al. 2015). The tomato fruit represents an open system into which additional sugars and amino acids can be imported in times of increased metabolic demand (increased sink strength). This means that switching on metabolic pathways in fruit, late in ripening (as conferred by the fruit-specific *E8* promoter, for example) can result in high levels of accumulation of metabolites without yield penalties, because fruit set, development and ripening are largely completed by this point (Butelli et al. 2008; Luo et al. 2008; Zhang et al. 2015; Scarano et al. 2017). Because tomato is such a good system for metabolic engineering, many isogenic lines enriched in different polyphenols are available for comparative nutrition experiments. In addition, lines important for metabolic engineering, such as the *E8:AtMYB12* line that induces primary metabolism (glycolysis, the TCA cycle, the pentose phosphate and the shikimate pathways) as well as flavonoid biosynthesis specifically in fruit, are already available and well characterised (Luo et al. 2008; Zhang et al. 2015).

Like other models, methods for stable and transient expression/silencing of target genes in tomato are well developed (Potrykus 1991; Fischer 1999; Hannon 2002; Orzaez et al. 2009). Recently, with the emergence of the new breeding technologies of genome editing, new alleles can be created, directly into the desired genetic background, to supply beneficial quantitative variation for tomato breeding (Rodríguez-Leal et al. 2017). CRISPR/Cas9 genome editing appears to be particularly efficient in tomato (Belhaj et al. 2015; Brookes et al. 2014; Pan et al. 2016).

Compared to other model plants, there are several unique research tools that have been developed to facilitate tomato research: a specialized variety, MicroTom, has a shorter growth cycle and reduced plant size. This cherry tomato variety can be used for fundamental research before traits are transferred to large-sized, globe tomato varieties (Dan et al. 2006). Use of the ethylene-inducible *E8* promoter ensures the expression of transgenes is induced only in ripe fruit. The *E8* promoter can be used in a general strategy to produce desirable compounds in fruit without yield penalties (Bovy et al. 2002; Luo et al. 2008; Zhang et al. 2015). In addition, the establishment of the *S. lycopersicum* × *S. pennellii* introgression lines (ILs) [and now, other IL populations including *S. lycopersicum* × S. *lycopersicoides, S. lycopersicum* × *S. pimpinellifolium, S. lycopersicum* × *S. sitiens, S. lycopersicum* × *S. chilense*, and *S. lycopersicum* × *S. habrochaites* (*S. hirsutum*)] has provided unique genetic resources to identify loci controlling important traits in tomato (Zamir 1995, 2001; Eshed and Zamir 1995; Frary et al. 2000; Fridman et al. 2000; Kushibiki and Tabata 2005; Powell et al. 2012).

## Metabolic engineering

Metabolic engineering is used to increase the accumulation of target metabolites in organisms. Metabolic engineering can be achieved by breeding of selective genotypes but more usually involves genetic engineering. Recent advances in genome editing are making this technique an additional option for many traits in tomato.

Most principles of metabolic engineering have been established in microbial systems, even for the production of plant natural products (Liu et al. 2017a, b) although there is increasing interest in using plants as chassis (O’Neill and Kelly 2016), especially for the development of nutritionally enhanced foods. The principles of metabolic engineering of plant natural products in microbes involve ensuring that each enzyme of the pathway is expressed, that each enzyme has optimized activity, that the flux along the pathway is selectively elevated, and that competing and catabolic pathways are blocked (Liu et al. 2017a, b). While these principles also hold true in plant metabolic engineering, the tools available to ensure that these design principles are met, are different in plants to those in heterologous microbial hosts. Originating from attempts to engineer lipid metabolism for the accumulation of oils, the terms ‘push’, ‘pull’ and ‘protect’ have been used to describe different engineering strategies (Van et al. 2014; Vanhercke et al. 2014). ‘Pull’ involves up-regulating the activities of enzymes that make the target molecule, particularly ‘key, rate-limiting’ steps in the biosynthetic metabolic pathways. Such approaches have been used very extensively in plant metabolic engineering, and usually provide modest increases in target metabolite content (Martin 1996; Farré et al. 2014). A good example is the enhanced production of flavonols in tomato resulting from ectopic expression of chalcone isomerase (Muir et al. 2001). ‘Protect’ strategies involve reductions in flux through pathways that compete for substrate or intermediates on route to the target molecule, or removing catabolic pathways that limit the accumulation of the target metabolite. Protect strategies have proved effective in folate biofortification of rice and in provitamin A engineering in sorghum (Blancquaert et al. 2015; Che et al. 2016). ‘Push’ strategies encompass those that increase flux along the biosynthetic pathways including activating transcription factors (TFs), as well as strategies that increase the supply of precursors from primary metabolism (Martin 1996; Butelli et al. 2008; Century et al. 2008; Luo et al. 2008; Fu et al. 2018). Generally, push strategies involve the use of transcriptional activators in plants that induce specific pathways, but recently a new type of transcriptional activator that can induce pathways of primary metabolism as well as those of secondary metabolism has been added to the tool-box. These TFs increase flux by supplying increased levels of substrates from primary metabolism, as well as energy and reducing power. Examples are: MYB12 (Zhang et al. 2015), WRI1 that activates fatty acid biosynthesis (Maeo et al. 2009; Baud et al. 2010; Marchive et al. 2015) and GAME9 from tomato that upregulates the MEP pathway to supply isopentyl phosphate precursors for terpenoid and sterol biosynthesis (Cárdenas et al. 2016). Although these activities may have been demonstrated originally in other species, all these tools are available for metabolic engineering in tomato (Fu et al. 2018).

## Tomato: an excellent biosynthetic chassis

**Fig. 1:**
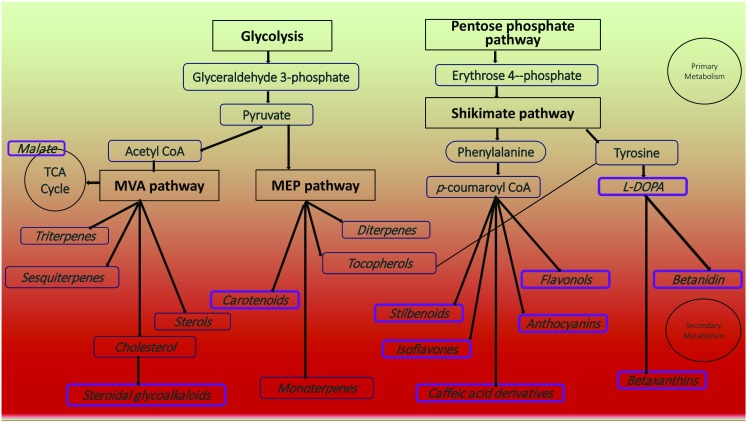
Important primary and secondary metabolites and their biosynthetic pathways in tomato fruit. Compounds that have been engineered already in tomato fruit are outlined in purple

Tomato is the world’s favorite fruit due to its special flavor and high nutritional value. Tomato fruit contains large amounts of metabolites such as sucrose, hexoses, citrate, malate and ascorbic acid. There are also many health-beneficial compounds such as carotenoids, phenylpropanoids and terpenoids that accumulate in tomato fruit (Fig. 1; Siddiqui et al. 2015). The existence of these compounds establishes that many basic biosynthetic pathways are intact in tomato. Therefore, when undertaking metabolic engineering, a limited number of additional genes needs to be introduced, which can significantly simplify the engineering process. In addition, substrates (such as sugars and aromatic amino acids) and intermediates (such as 4-coumaroyl CoA and acetyl-CoA) that are needed for secondary metabolism are often enriched in tomato fruit (Fig. 1). All these features facilitate the use of tomato fruit as a chassis for metabolic engineering.

So far, the best examples of metabolic engineering in tomato involve the phenylpropanoid pathway. Phenylpropanoids arise from the essential amino acid phenylalanine and *p*-coumaroyl CoA produced from phenylalanine by the general phenylpropanoid pathway (Fig. 1; Vogt 2010). Tomato fruit contain various phenolic compounds (flavonoids, caffeoyl quinic acids and other hydroxycinnamates) which show that the phenylpropanoid biosynthetic network is intact and active in fruit and can be engineered to either enhance the production of existing phenylpropanoids or produce new types of compound. Over-expression of genes encoding purported ‘rate-limiting steps’ in the phenylpropanoid pathway was first used to induce biosynthesis, particularly of flavonoids (Muir et al. 2001; Vogt 2010; Tzin et al. 2013). Several MYB and bHLH transcription factors (TFs) have been shown to induce the expression of phenylpropanoid biosynthetic genes. Overexpression of these TFs in tomato fruit can significantly enhance the production of phenylpropanoids (Bovy et al. 2002; Broun et al. 2006; Luo et al. 2008; Butelli et al. 2008; Gonzali et al. 2009; Zhang et al. 2015). Introduction of new structural genes encoding enzymes into tomato can create new compounds such as resveratrol and genistin from current biosynthetic pathways (Schijlen et al. 2006; Carrillo et al. 2011; Zhang et al. 2015). Recently, using the *Arabidopsis* transcription factor AtMYB12, we managed to switch on aromatic amino acid biosynthesis by manipulating primary metabolism. The AtMYB12 protein not only efficiently induces production of phenylpropanoid compounds, but also has the potential to induce the production of high-value metabolites derived from tyrosine and tryptophan in tomato (Zhang et al. 2015).

## Examples of high-value metabolites produced in tomatoes

Betalains, one of the three major types of pigments in plants, provide the colors seen in fruits and flowers of some members of the family Caryophyllaceae. Betalains have been used extensively as natural colorants for many centuries (Georgiev et al. 2008). They are tyrosine-derived, red–violet and yellow pigments used as food colorants and dietary supplements, which are generally classified into the red betacyanins and the yellow betaxanthins (Schwinn et al. 2016). Metabolic engineering for heterologous betalain production was achieved for the first time in tomato, following expression of three genes encoding the cytochrome P450 CYP76AD1, the BvDODA1 dioxygenase, and the cDOPA5GT glycosyltransferase in a single binary vector. As much as 248 ± 41 mg L^− 1^ betalain were produced in tomato juice. In addition, these lines have been crossed with a *Del*/*Ros1* tomato line with elevated anthocyanin production, which can further increase the content of betalain in fruit (Butelli et al. 2008; Polturak et al. 2017).

Recently, tomato fruit have been engineered to produce ketocarotenoids. Ketocarotenoids, such as canthaxanthin, adonirubin, or astaxanthin are high-value pigments used commercially across the food and feed industries, although they are rarely synthesized in plants. This engineering strategy involved both the enrichment and the extension of the β-carotene pathway. The genes encoding β-carotene hydroxylase (*CrtZ*) and the oxyxgenase (*CrtW*) from *Brevundimonas* sp. as well as the allele encoding the lycopene β-cyclase (*β-Cyc*) from *Solanum galapagense* were introduced into tomato fruit. Two independent aquacultural trials identified that the plant-based feeds developed were increased in the retention of the main ketocarotenoids twofold, in the fillets of fish fed on ketocarotenoid-enriched feed compared to control feed (Nogueira et al. 2017).

## New high value compounds can be produced by tomato

Based on our understanding of the metabolic networks active in tomato fruit and previous metabolic engineering studies, other bioactive compounds that could be produced successfully in tomato can be suggested.

For the biosynthesis of Rosmarinic Acid (RA), there are two precursors, l-phenylalanine (which is converted to *p*-coumaroyl-CoA, catalyzed by the enzymes of the General Phenylpropanoid Pathway, phenylalanine ammonia lyase, cinnamate 4-hydroxylase, and *p*-coumaroyl CoA ligase) and l-tyrosine which is converted to 4-hydroxyphenyllactic acid, catalyzed by tyrosine aminotransferase (TAT) and 4-hydroxyphenylpyruvate reductase (HPPR), enzymes which are active in tomato fruit. The activity of RA synthase produces 4-coumaroyl-4′-hydroxyphenyllactic acid, and then the 3- and 3′-hydroxyl groups are introduced by a cytochrome P450 monooxygenase to produce RA (Ru et al. 2016). Previous studies have indicated that AtMYB12 can enhance significantly the synthesis of aromatic amino acids (phenylalanine, tyrosine and tryptophan) (Zhang et al. 2015). Thus, co-expression of AtMYB12 and additional structural genes could be used to produce substantial amounts of RA in tomato.

Pharmacological studies have shown that retinol (vitamin A) is essential for the development of the human central nervous system (CNS). Retinol can help resist Parkinson’s disease and Alzheimer’s disease (Kunzler et al. 2017; Liu et al. 2017a, b; Sato et al. 2017). In tomato, enhancing the levels of provitamin A can be achieved by manipulating β-carotene biosynthesis. β-carotene concentrations can be improved either by increasing synthesis or reducing catabolism. Lycopene, with high lipophilic antioxidant capacity, is the red compound that accumulates in ripe tomatoes, and lycopene can be metabolized to α-carotene or β-carotene. Therefore, selection of weaker alleles of the gene encoding lycopene ε-cyclase (*LcyE*), an enzyme that transforms all-*trans* lycopene into δ-carotene, has been shown to enhance the concentration of β-carotene. Alternatively, weakening the expression of the gene encoding β-carotene hydroxylase (*HydB*), which converts β-carotene to zeaxanthin, can increase β-carotene levels in tomato by a ‘protect’ strategy. β-carotene can be converted to two molecules of retinol, meaning that β-carotene is a better source of provitamin A than other carotenoids which can give rise to only one molecule of retinol. Overexpression of the gene encoding carotene ε-ring hydroxylase (*CYP97C*), turns α-carotene into lutein which is richest in green leafy vegetables (such as spinach, broccoli, peas and lettuce), and protects against the development of age-related macular degeneration (AMD), due to its selective accumulation in the macula of the retina of the eye. β-carotene improves visual function in patients with age-related cataracts and non-proliferative diabetic retinopathy (Olmedilla et al. 2003; Zhu et al. 2010; Zhang et al. 2017).

There are two forms of vitamin E vitamers (tocopherols and tocotrienols) collectively defined as tocochromanols in most plants. The bioavailability of tocochromanols is dependent on their affinity for the α-tocopherol transporter in the liver of humans, and tocochromanols protect against low density lipoprotein (LDL) and polyunsaturated fatty acid (PUFA) oxidation, cardiovascular disease, some cancers and impaired immune function (Martin and Li 2017). Tocotrienols are not produced in tomato because of the absence of a gene encoding homogentisate geranylgeranyl transferase (*HGGT*) in tomato (Lu et al. 2013). Screening for stronger alleles of the gene encoding homogentisate phytyl transferase (*HPT; vte2*) could increase significantly the concentrations of tocopherols in tomato (Mène-Saffrané and Pellaud 2017).

Cholesterol and its derivatives are precursors for thousands of important compounds including: the steroidal saponin, diosgenin, which serves as a hormonal drug as well as its derivative progesterone; the steroidal alkaloid (SA) solamargine, which serves as potential cancer drug as well as pro-vitamin D3, which is also known as 7-dehydrocholesterol (Sonawane et al. 2016). SAs and their glycosylated forms (steroidal glycoalkaloids; SGAs) are nitrogen-containing toxic compounds occurring primarily in the Solanaceae and Liliaceae plant families. Although SGAs confer resistance of *Solanaceous* species to a comprehensive list of pathogens and predators, some are regarded as anti-nutritional compounds for humans including α-tomatine and dehydrotomatine in green tissues of fruit (Itkin et al. 2013; Sonawane et al. 2016). The elucidation and manipulation of the cholesterol pathway in tomato could be a first step towards plant-based engineering of interesting cholesterol derivatives. One of the steroidal alkaloids, dioscin, is the main bioactive component of *Dioscorea nipponica* Makino tubers, and has been used as a marker compound for evaluating the quality of *Dioscorea nipponica* Makino in traditional Chinese medicines (Yin et al. 2010). Dioscin is an essential feed stock for the steroidal hormone industry, and because it has the same carbon skeleton as SGA, it could be produced in tomato.

## Scale-up of production in tomato

**Fig. 2:**
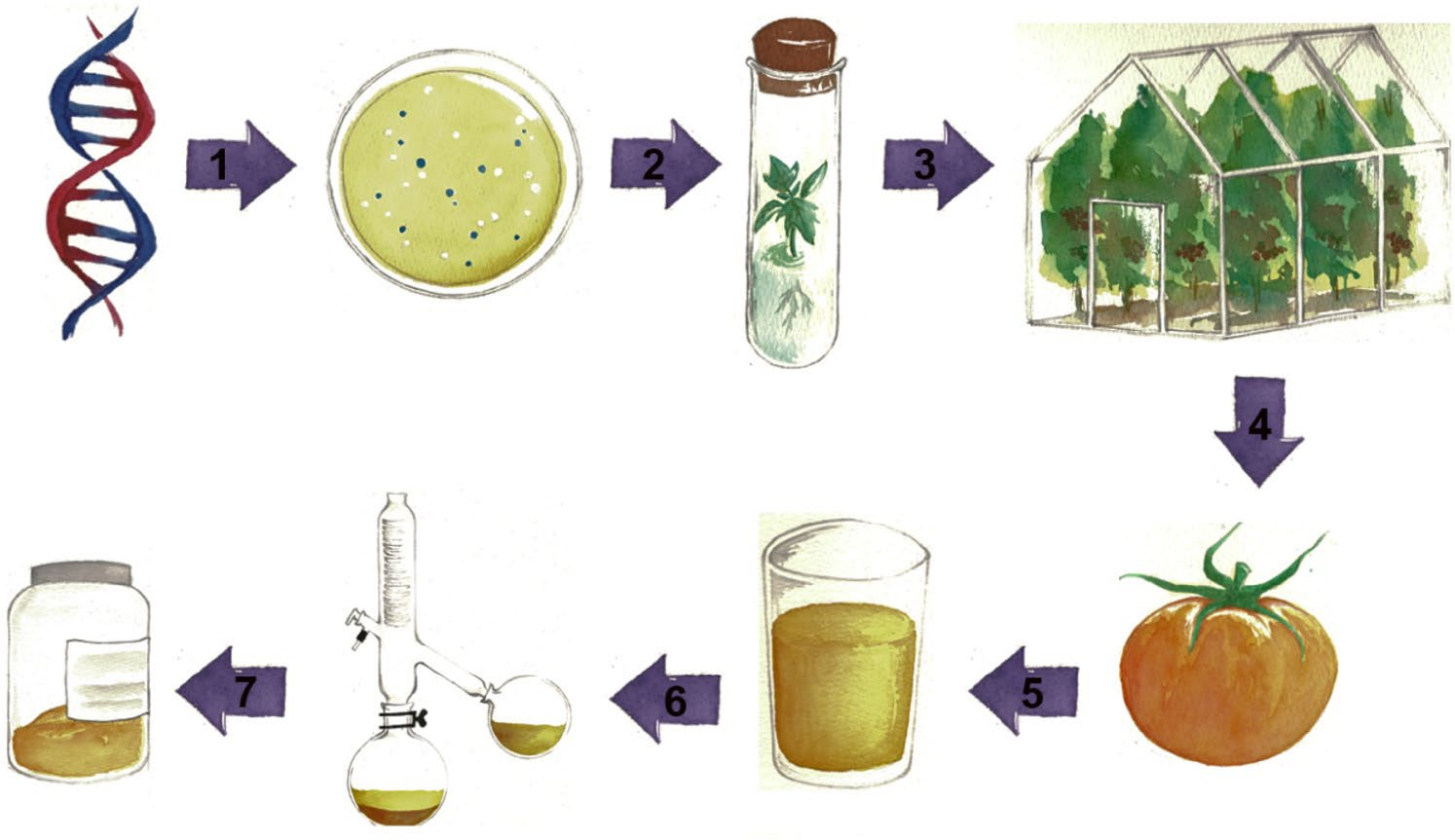
Production of valuable metabolites using the proposed tomato production platform. Using metabolic engineering, valuable compounds can be produced in tomato fruit. The engineered tomato plants could be grown in containment greenhouses. Fruit juice could be processed to produce metabolite extracts and downstream facilities could be used to purify target compounds. (1) Construction of vectors for metabolic engineering in tomato fruit; (2) agrobacterium-mediated transformation of tomato to produce engineered fruit; (3) multiplication of engineered lines for cultivation in containment (insect-proofed) greenhouses; (4) harvesting of fruit; (5) preparation of extracts of high value chemicals from tomatoes. This may be as simple as homogenization and centrifugation to generate ‘tomato water’ for high value, water soluble compounds; (6) chemical separation methods for purification of high value compounds; (7) sale of high value metabolite products from tomato

To assemble a successful production platform, a controlled space for cultivation of genetically engineered plants needs to be established, unless field cultivation has been granted regulatory approval. Containment can be accomplished using insect-proofed greenhouses for cultivating tomatoes. The end product of this process could be tomato juice containing the target metabolites. Seeds could be removed during juicing to avoid any potential environmental impact. Plant waste and pumice can be devitalized by autoclaving or incineration (Fig. 2).

A second strategy involves adaptation of any new production system to current industrial processes. A tomato production system could be divided into two major parts: the production of tomato juice and the purification of desired compounds (Fig. 2). The first part could be adapted to current methods used in the juice production industry, where equipment and protocols have already been optimised to remove seeds and concentrate juice. The only difference would be the replacement of non-GM field-grown tomatoes with fruit from engineered plants cultivated in containment greenhouses. For the second stage, tomato juice could be processed further to produce high-purity compounds. This stage could be readily adapted from existing microbial production platform purification protocols. In such cases, tomato juice containing high-value metabolites would replace the microbial medium in purification protocols. All techniques used for purification of metabolites from microbial medium should be similarly applicable to tomato juice. To summarize, tomato production platforms could readily be developed based on existing infrastructure for glasshouse cultivation of fresh market tomatoes coupled to existing industrial purification platforms by combining practices from the tomato juice industry with microbial production systems (Fig. 2).

## Conclusions

Tomato offers a useful chassis for metabolic engineering, with significant advantages over other chassis: (a) it is high yielding, easy to grow and manage with existing tomato cultivation infrastructure; (b) fruit contain most of the necessary substrates; (c) fruit contain the whole or most of the biosynthetic pathways for making high-value metabolites and activity can be further enhanced by engineering the activity of transcription factors (Fu et al. 2018); (d) genome sequence is available with many additional tools and resources that facilitate metabolic engineering; (e) fruit-specific production of secondary metabolites usually does not incur a yield penalty nor affect the growth of the plant. Tomato should be considered more frequently for sustainable production of high-value specialty metabolites.

### Author contribution statement

YL, YZ and CM conceived and co-wrote this review. HW drew Fig. 2.
